# Assembling unregulated DNA segments bypasses synthesis screening: regulate fragments as select agents

**DOI:** 10.1038/s41467-025-67955-3

**Published:** 2026-01-15

**Authors:** Rey Edison, Shay Toner, Kevin M. Esvelt

**Affiliations:** 1 Microbiology Program, Massachusetts Institute of Technology, Cambridge, MA USA; 2 Media Lab, Massachusetts Institute of Technology, Cambridge, MA USA

**Keywords:** Policy, Policy and public health in microbiology, Synthetic biology

## Abstract

U.S. select agent regulations ignore easily assembled DNA fragments, making synthesis screening ineffective regardless of accuracy. We acquired unregulated DNA collectively sufficient for a skilled individual to generate 1918 influenza from dozens of providers, demonstrating that fragments must be regulated as select agents.

Current U.S. regulations governing select agent pathogens contain a critical loophole: they cover intact DNA sequences capable of generating harmful agents^1^, but ignore DNA fragments that can be assembled by anyone with modest laboratory skills (Fig. 1). Under this flawed system, providers have no legal obligation to monitor or restrict access. To explore this vulnerability, we fragmented DNA sequences encoding ricin toxin or 1918 pandemic influenza virus and ordered 2-8 pieces from each of 38 DNA synthesis providers^2^. The companies collectively provided enough (legal) 400–500 base-pair fragments to generate (illegal) intact constructs several times over. Laboratory tests of equivalent harmless sequences confirmed that the pieces could have been assembled into plasmids that would enable a skilled individual following public protocols to generate a virus that once killed 50 million people^3^. Responsible firms face an impossible choice: implement voluntary safeguards and potentially lose business to less cautious competitors, or ship dangerous but legal fragments. Market incentives discouraged them from verifying our identity, confirming a legitimate research purpose, or coordinating with others to determine whether we sought to obtain an intact agent. The question is whether policymakers will update select agent regulations to cover DNA fragments and enforce compliance through mandatory stress-testing, as in cybersecurity^4^, to level the playing field and prevent misuse.

**Fig. 1:**
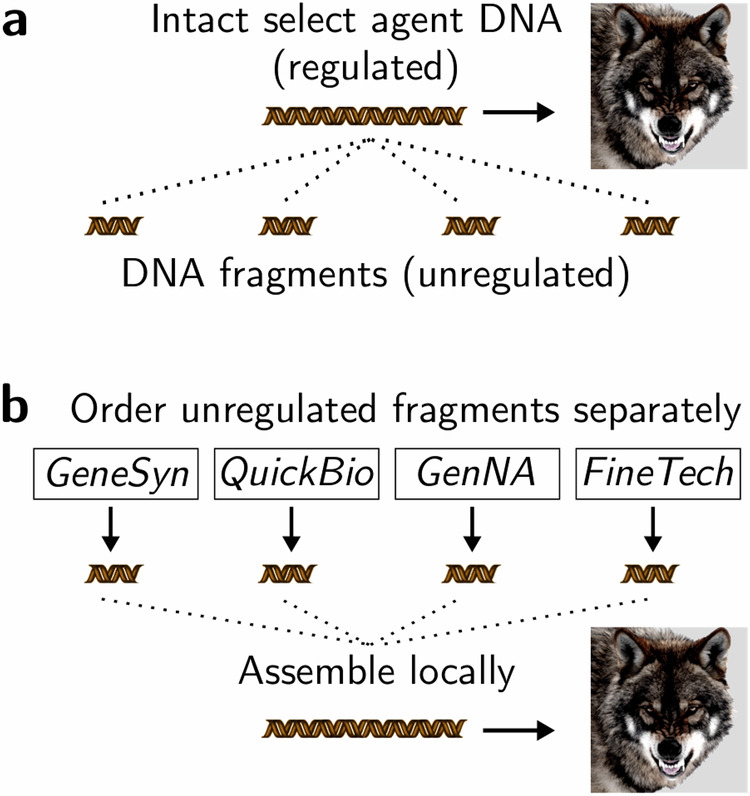
Splitting select agent DNA into unregulated fragments for subsequent assembly bypasses controls. **a** Intact DNA sequences that can be used to produce weaponizable select agents (wolf icon) are regulated, but fragments are not. **b** Legal, individually harmless fragments of select agent DNA ordered from separate DNA synthesis providers who are unaware that other companies are providing the remainder can be locally assembled using common techniques to bypass select agent controls. Wolf image: AI-generated, Adobe Stock, unlimited web use.

Regulating synthetic DNA fragments is feasible: freely available screening systems can reliably detect controlled sequences with very few false alarms and negligible delays^5,6^, while commercial systems offer additional protection^7,8^. However, current regulations do not require their use. France, which recently alerted providers that their regulations govern certain pieces of DNA from select agents over 500 nucleotides^9^, is the only nation with such controls – leaving DNA synthesis providers elsewhere with little incentive to screen potentially dangerous sequences. Instead, firms are advised to voluntarily screen and verify orders ^10^.

Members of the International Gene Synthesis Consortium (IGSC) have demonstrated good faith by screening customer sequences for nearly two decades at their own expense, even as the cost has grown^11^. But as we heard again and again upon reaching out to companies after the experiment, they cannot delay orders to confirm that a responsible third party has verified the research relevance of every individually harmless (and entirely legal) DNA fragment without losing customers to less responsible firms.

This is particularly concerning for select agent viruses such as 1918 influenza. The virus is unlikely to cause a pandemic today because modern related strains provide some cross-immunity^12,13^, but a mere 10% probability of a new pandemic would kill several million people in expectation – a total rivaling that of a nuclear detonation. The variola virus responsible for smallpox would cause a more devastating pandemic if synthesized and released, but is extraordinarily challenging to obtain due to its very large genome and the much greater difficulty of producing infectious poxviruses from synthetic DNA.

As well-intentioned labs seek to credibly identify other viruses likely to cause new pandemics^14^ and to publicly release machine learning models capable of predicting which mutations in existing viruses could create omicron-like immunity-escaping variants, the risk of misuse will grow^15^. Large language models can suggest fragmentation and evasion strategies to users seeking to circumvent synthesis screening, as well as answer questions relevant to troubleshooting virology protocols^16^, further lowering technical barriers for potential misuse. In short, we are likely to transition from the current world – where there is little chance of a deliberate pandemic because people don’t know of any accessible pandemic-capable viruses – to one in which many sets of pandemic blueprints are publicly available. Before that happens, the international community needs to restrict access to DNA that could be used to generate pandemic pathogens.

## Assessing DNA synthesis screening for fragments of select agents

**Fig. 2:**
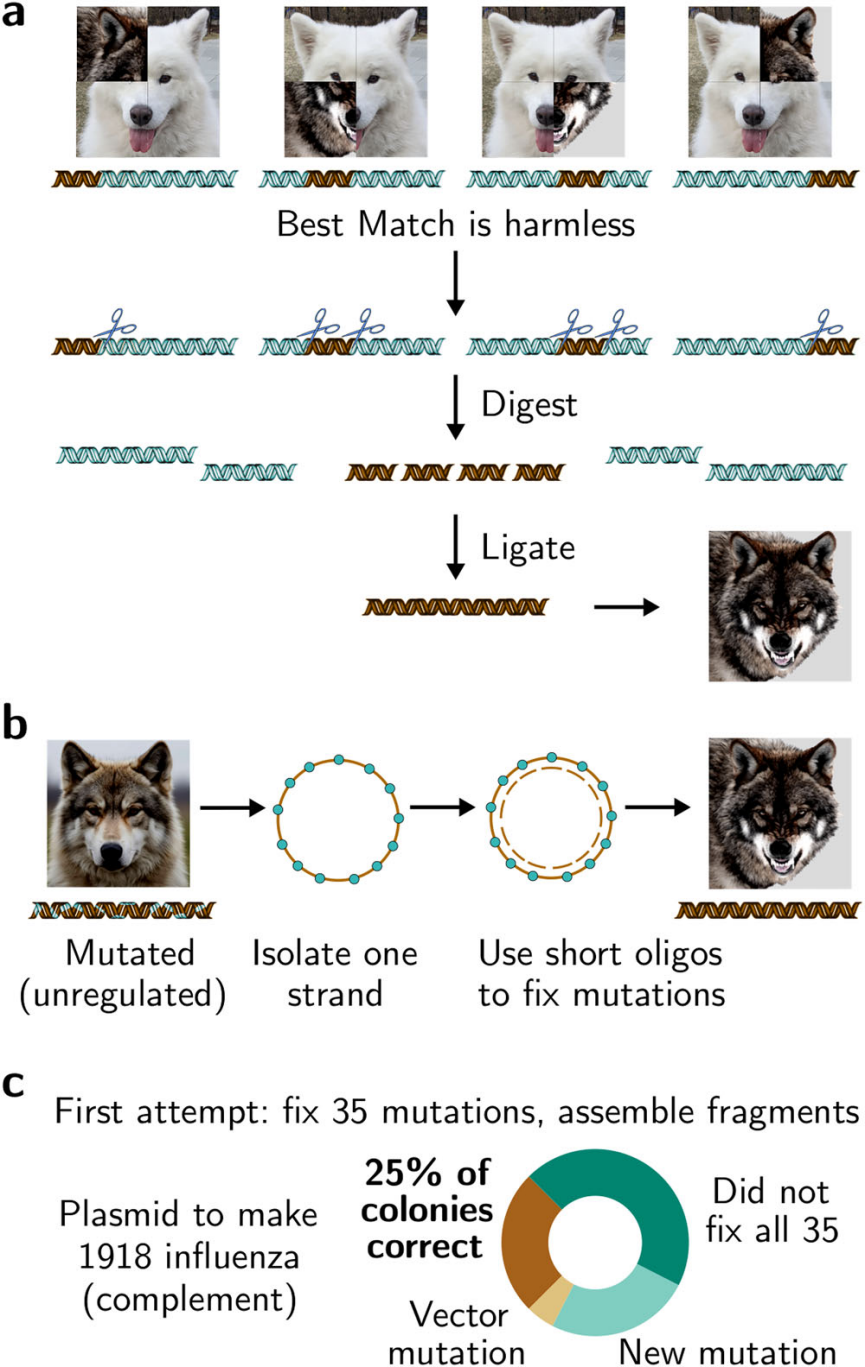
Screening must detect camouflaged split orders. **a** Legal fragments can be camouflaged to evade whole-sequence screening by adding a harmless related sequence (dog icon), then subjected to standard Golden Gate assembly. 24/25 non-IGSC providers each shipped two to nine legal, camouflaged fragments of 1918 influenza virus; 8/8 also shipped legal fragments of ricin toxin. Following reported law enforcement contact to discuss the results, 8/8 shipped undisguised legal fragments of 1918 influenza. **b** Intact DNA sequences with introduced mutations are not regulated, may escape screening, and can be corrected using short DNA pieces that exactly match the select agent sequence but are neither regulated nor detected by screening guidelines^18^. 12/13 additional providers (beyond the 25 above) shipped legal, mutated fragments of 1918 influenza virus, 5/6 of which also shipped ricin fragments. **c** Fraction of sequence-confirmed colonies resulting from the simultaneous correction of 35 mutations using oligonucleotides shorter than 50 base-pairs followed by the assembly of fragments complementary to 1918 influenza by a graduate student who had never previously performed a Darwin Assembly protocol^18^. The high success rate underscores the need to regulate and screen all synthetic DNA fragments over 30 base-pairs for their capacity to generate select agent viruses. Samoyed photo: author’s own. Mutated image: blend.

To test whether current voluntary screening policies can prevent the acquisition of U.S. select agents, we carefully designed fragments of 1918 influenza and ricin toxin to be detectable only by providers using screening compliant with then-current U.S. guidance^10^, employing camouflage and introduced mutations (Fig. 2a, b). We deliberately did not employ these methods to their fullest extent, instead ensuring that assessing every 200 base-pair subsequence per the guidance would suffice for detection. Upon discovering a sequence of concern, the guidance advised providers to contact the biosafety officer of the relevant institution to verify the customer’s legitimate research need for the associated gene, toxin, or agent^10^. We placed orders with 38 providers from companies located in nations approved by U.S. government officials and with the explicit awareness of law enforcement, then counted legitimacy requests and shipments received.

While our strategy could not determine whether most providers failed to detect the controlled sequences or detected them but chose not to verify research legitimacy, it directly assessed whether voluntary regulations are sufficient to restrict access to a select agent pandemic virus.

Despite using a pseudonym with no molecular biology publications and providing an office shipping address and contact details for a desk-based nonprofit with no laboratory facilities, we obtained fragments encoding both ricin toxin and 1918 influenza virus from nearly all providers without registering with the Federal Select Agent Program (FSAP) or triggering any notifications of law enforcement. For context, research institutions in possession of select agents or intact DNA sequences sufficient to generate them must register with the FSAP, demonstrate proper facilities and safety protocols, undergo personnel screening, and maintain strict inventory controls. That anyone can legally obtain the DNA required to produce these same agents by ordering DNA fragments represents a critical failure in the regulatory framework.

To verify that these fragmented select agent orders could be successfully assembled without ourselves generating dangerous or illegal constructs, we ordered complementary camouflaged or mutated fragments that behave identically in molecular biology protocols, but cannot produce viral proteins. The complementary fragments were readily assembled using Golden Gate, one of the most widely used molecular cloning methods^17^. The complementary mutated fragments, which we had disguised by incorporating at least one mutation every 48 base-pairs, were corrected using a standard method for targeted genetic variant creation^18^ that employs oligonucleotides shorter than 50 base-pairs: all 35 mutations were fixed with a high success rate on the first attempt (Fig. 2c).

In the case of viral agents, assembling the correct plasmids is insufficient to cause harm: there remains the challenge of producing infectious viruses. However, reverse genetics protocols for many viral families are readily available, if more challenging to complete successfully than the Golden Gate or Darwin Assembly techniques used here. That most individuals capable of reverse genetics are likely capable of assembling DNA fragments using our methods demonstrates the vital importance of screening oligonucleotides shorter than 50 base-pairs, which otherwise can be used to transform innocuous sequences into related select agents^18^ or to assemble them directly via Golden Gate^17^. Collectively, our results demonstrate that modern molecular biology techniques can readily bypass current laws by assembling legal fragments of DNA encoding select agents.

Several weeks after our law enforcement contacts indicated they would have spoken with each provider, we conducted a subsequent stress-test to assess whether eight domestic non-IGSC firms had addressed the vulnerability. All eight shipped completely undisguised 1918 influenza fragments. Not one requested authorization, verified our identity, or reported the suspicious attempt to authorities, demonstrating the fundamental inadequacy of voluntary compliance.

In contrast, multiple IGSC firms reached out to explain that they had detected controlled sequences in our initial orders, but shipped the fragments because they were legal and harmless individually. They have no way to detect orders split between multiple providers. Several noted that they cannot voluntarily request authorization without losing customers to less cautious firms, demonstrating how the current regulatory gap creates market pressures that actively undermine responsible providers and national security.

Of the 36 total providers that shipped legal select agent DNA fragments, only one requested evidence of authorization from a third party. That firm requested verification from a biosafety officer because it detected ricin, which we had designed to be discoverable even by certain forms of sub-standard screening that only check the entire order rather than every 200 base-pair window. But they shipped fragments of 1918 influenza that were exclusively detectable by guidance-compliant screening without comment. In short, even the firm apparently most willing to defy market pressures for the greater good raised no concern over 1918 influenza, suggesting that they could still benefit from third-party stress-testing to identify vulnerabilities in their security measures.

Collectively, our results reveal how inadequate select agent regulations prevent even responsible DNA synthesis providers from consistently implementing vital safeguards. Synthetic DNA sufficient to generate infectious 1918 influenza virus following standard laboratory assembly can be purchased for approximately $3000; we obtained enough fragments to encode the virus several times over. As a result, anyone with modest laboratory skills and access to standard equipment can order and assemble DNA fragments sufficient to generate select agent viruses and toxins – including a pandemic virus that killed over 50 million people.

Consistent with cybersecurity norms, we delayed public disclosure for over 90 days after privately notifying providers of our findings and the availability of a free, privacy-preserving screening system capable of detecting each of the strategies we demonstrated – even when employed to their fullest extent using oligonucleotides shorter than 50 base-pairs^5^. Following our experiment, some commercial screening system operators indicated that they would update their systems; these services could also address split-orders if they could share partial matches to regulated sequences without creating privacy issues. Unfortunately, widespread adoption and adherence to security screening protocols by oligonucleotide and gene synthesis providers – as well as synthesis device manufacturers – will require policy reform.

## Policy Recommendations

Legal mandates demonstrably drive industry adoption where voluntary measures fail. Following the 2024 publicization of France’s regulation of DNA fragments over 500 nucleotides corresponding to select agents^9^, adoption of screening tools increased markedly and specifically among providers serving French researchers – the same providers who had previously declined to voluntarily adopt even free, privacy-preserving tools. This natural experiment underscored that legal requirements with criminal liability can overcome the market failures preventing security improvements.

Our experiments highlight the existence of legal loopholes across virtually all jurisdictions, even France: because we ordered fragments shorter than their 500 base-pair minimum^9^, unregulated pieces can be ordered from multiple providers, assembled, and used to generate dangerous pathogens such as the 1918 influenza virus in any nation. Export controls do not prevent domestic acquisition and apparently offer little protection against international orders: most international providers were located in signatories of the Australia Group export control treaty regulating ricin and 1918 influenza, but shipped DNA anyway, perhaps because it is unclear whether language specifying ‘genetic elements that contain nucleic acid sequences associated with pathogenicity’ or ‘coding for any of the toxins specified’ are applicable to fragments.

As a simple and immediate international fix that would not require any legislative action in many jurisdictions, we propose that nations should update their select agent regulations to cover short fragments of select agent genes, accompanied by lighter access requirements. In the United States, for example, this could be accomplished by regulating fragments as lower-security Tier III agents requiring straightforward electronic registration before purchase. Select agent viruses regulated by the Department of Health and Human Services (HHS) offer a logical starting point for three reasons: their ability to spread autonomously once generated from synthetic DNA renders them capable of causing far more harm than toxins could, they represent a well-defined set for regulation, and they do not tolerate extensive changes across their genome sequences that might otherwise allow redesigned toxins or individual proteins to evade screening^19^. Even careful fine-tuning of generative models trained on bacteriophage genome data generated functional phages that were at least 93% similar to natural sequences^20^, whereas similarity under 60% is typically needed to evade screening.

Any effective regulation of select agent DNA must include DNA oligonucleotides shorter than the 50 base-pair screening window specified by U.S. guidelines released in mid-2024, which would fail to detect the 30–49 base-pair oligonucleotides we used to correct camouflaging mutations and did not mandate screening sufficient to detect camouflaged sequences (Fig. 1d). We accordingly recommend that any set of short DNA fragments (e.g. over 30 bases each) matching a controlled sequence that collectively span a significant fraction of a typical gene length (e.g. 200 or more total bases) be regulated as a Tier III agent. Crucially, existing congressional legislation appears to provide the HHS Secretary with unilateral authority to make all of these changes for HHS select agent viruses^21^, without involving the Department of Agriculture or other agencies. Implementation can occur in three stages:

### Stage 1 (registration)

To simplify legitimate research access, update the existing eFSAP registration portal to allow straightforward registration of specific DNA fragments, removing unnecessary burdens associated with existing higher-tier agents. Researchers should be able to simply log in, enter the sequences they need, and either describe the research goal or provide an already-approved institutional biological research registration document, avoiding the cost and bureaucracy of research with Tier I or Tier II agents.

### Stage 2 (verification)

To enable efficient compliance, establish an automated system for DNA providers and benchtop DNA synthesizer manufacturers to instantly verify that a request for regulated fragments has been properly registered by an authorized user. SecureDNA has already implemented a cryptographic “public key infrastructure certificate” system for this purpose^5^ and has been working with other organizations to develop an industry standard based on the prototype; the eFSAP portal could simply issue these certificates to be uploaded alongside synthesis orders.

### Stage 3 (tracking)

To prevent attackers from bypassing controls by splitting orders across different companies or countries, implement a global tracking mechanism – potentially adopting the free privacy-preserving Switzerland-based system^5^ to perform this function on top of any existing safeguards – to monitor the synthesis of regulated fragments across national boundaries. Since Chinese providers that were not evaluated by our experiment are estimated to account for 40% of the global market, their eventual participation will be essential for global security.

Lastly, FSAP can clearly define exactly which subsequences from the HHS select agent viruses will be treated as Tier III agents, and require DNA synthesis providers and synthesis device manufacturers to deny unregistered access. Compliance should be verified through regular stress-testing against standards determined in consultation with experts in biosecurity screening, including attempts to order pieces from providers across different borders. Once the system has been operating smoothly for several months, biosecurity experts can be convened to develop standards for identifying other sequences posing similar risks.

We recommend that nations lacking select agent programs move to establish them, including DNA fragments, and pass legislation mandating stress-testing. While current select agent lists are incomplete, woefully neglect the importance of transmissibility, and require supplementation to include functional equivalents, capability-based restrictions should be grounded in known agents.

Implementing these policy changes is essential because even though technical solutions exist, voluntary measures have proven insufficient: current regulations create a market where security compromises are effectively rewarded, allowing easy acquisition of DNA capable of generating history’s deadliest pathogens. Open discussion is necessary to spur adoption of effective safeguards, especially as the developers of emerging AI tools for biological design are explicitly assuming that superior protections will be operational ^22^.

Replacing the failed voluntary system with explicit legal requirements, starting with HHS select agent viruses, will be crucial to secure the life sciences against potentially catastrophic misuse. That we legally obtained fragments of DNA collectively sufficient to generate a virus that once killed 50 million people from virtually every gene synthesis provider, without any questions being asked, represents a noteworthy failure of societal risk assessment and mitigation. The cybersecurity sector has shown that regular stress-testing – the use of third-party teams to discover and privately report potential vulnerabilities – can help prevent exploitation^4^. By updating FSAP to mandate verifiable screening across all modes of DNA synthesis while preserving legitimate research access, policymakers can prevent deliberate pandemics, and the draconian restrictions that would inevitably follow, while supporting continued advances in biotechnology and biomedicine.
